# Artificial intelligence predictive system of individual survival rate for lung adenocarcinoma

**DOI:** 10.1016/j.csbj.2022.05.005

**Published:** 2022-05-14

**Authors:** Tingshan He, Jing Li, Peng Wang, Zhiqiao Zhang

**Affiliations:** Department of Infectious Diseases, Shunde Hospital, Southern Medical University, Shunde, Guangdong, China

**Keywords:** Lung adenocarcinoma, Artificial intelligence, Prognostic model, Overall survival

## Abstract

**Background:**

The current research aimed to develop an artificial intelligence predictive system for individual survival rate of lung adenocarcinoma (LUAD).

**Methods:**

Independent risk variables were identified by multivariate Cox regression. Artificial intelligence predictive system was constructed using three different data mining algorithms.

**Results:**

Stage, PM, chemotherapy, PN, age, PT, sex, and radiation_surgery were determined as risk factors for LUAD patients. For 12-month survival rate in model cohort, concordance indexes of RFS, MTLR, and Cox models were 0.852, 0.821, and 0.835, respectively. For 36-month survival rate in model cohort, concordance indexes of RFS, MTLR, and Cox models were 0.901, 0.864, and 0.862, respectively. For 60-month survival rate in model cohort, concordance indexes of RFS, MTLR, and Cox models were 0.899, 0.874, and 0.866, respectively. The concordance indexes in validation dataset were similar to those in model dataset.

**Conclusions:**

The current study designed an individualized survival predictive system, which could provide individual survival curves using three different artificial intelligence algorithms. This artificial intelligence predictive system could directly convey treatment benefits by comparing individual mortality risk curves under different treatments. This artificial intelligence predictive tool is available at https://zhangzhiqiao11.shinyapps.io/Artificial_Intelligence_Survival_Prediction_System_AI_E1001/.

## Graphical abstract

**Figure:**
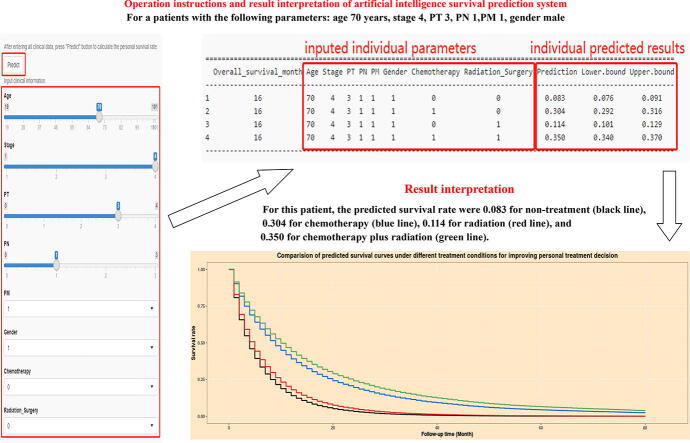

## 1 Background

Lung adenocarcinoma (LUAD) is one of the most common malignant tumours, accounting for 1.8 million cancer related deaths [1]. The prognosis of LUAD patients is still unsatisfactory until today [2]. At present, there were many predictive models in predicting survival rate for LUAD patients at the group level [3], [4]. However, the prognosis of LUAD patients with different clinical characteristics is complicated till now. Therefore, the prognostic prediction of one special group is far from meeting the need of individualized treatment decisions for a special individual patient.

In recent years, artificial intelligence has made great progress in cancer research, diagnosis, prognostic prediction, and treatment. Various algorithms have been used to find the lncRNAs closely related to different diseases, so as to provide valuable biomarkers for clinical diagnosis [5], [6], [7], [8]. Artificial intelligence predictive models based on gene expression data could predict prognosis for different tumors [9], [10]. Artificial intelligence algorithms based on gene expression data could also be used to predict the efficacy of tumor treatments [11], [12]. Tumor imaging recognition based on deep learning technology is helpful for early diagnosis and accurate classification for tumor [13]. The above researches suggested that artificial intelligence has broad application prospects in cancer research, diagnosis, prognostic prediction, and treatment.

From 2012 to 2013, Professor Gary S Collins developed several on-line prognostic predictive tools to predict mortality for different tumours [14], [15], [16], [17], [18]. In recent years, several studies have predicted individual survival curves for cancer patients based on different algorithms [19], [20], [21]. Our research team developed several precise medicine predictive systems to predict individual survival curves for different cancers before clinical treatment based on genetic data [22], [23], [24], [25], [26], [27], [28], [29]. Several experts proposed valuable suggestions for improving our precision medicine tools presented in our previous articles. Can precision medicine predictive tools provide individualized mortality curves for patients receiving radiotherapy or chemotherapy? Can the current precision medicine predictive tools convey treatment benefits by comparing the individual mortality curves of patients under different treatments, which might be more valuable for optimizing individual treatment decisions?

Therefore, according to previous suggestions, our team planned to develop an artificial intelligence predictive tool in predicting and compare individual survival curves of LUAD patients under different treatments.

## 2 Methods

### 2.1 Study cohorts

All datasets were obtained from the Surveillance Epidemiology and End Results (SEER) database (2010–2015). All included patients were diagnosed with lung adenocarcinoma (ICD-O-3 code: 8140). To eliminate the confounding effects of other causes, living subjects with survival time <12 months were removed from present study (n = 852).

### 2.2 Research methods

Induction and deduction are common research methods in scientific research [30]. The data type in the current study belongs to cohort study data. The current research used induction method to summarize cohort research information of LUAD patients, so as to obtain general rules of prognosis of LUAD patients. Then, the current research used the deductive method to study the prognosis of individual patients from the general rules of the overall cohort.

### 2.3 Artificial intelligence algorithms

The random survival forest algorithm was performed in accordance with the original studies [31], [32], [33], [34]. The multitask logistic regression (MTLR) algorithm performed in line with the suggestions of previous articles [35], [36]. The Cox proportional hazards algorithm was performed based on advices in original articles [37], [38].

### 2.4 Statistical analyses

**Fig. 1:**
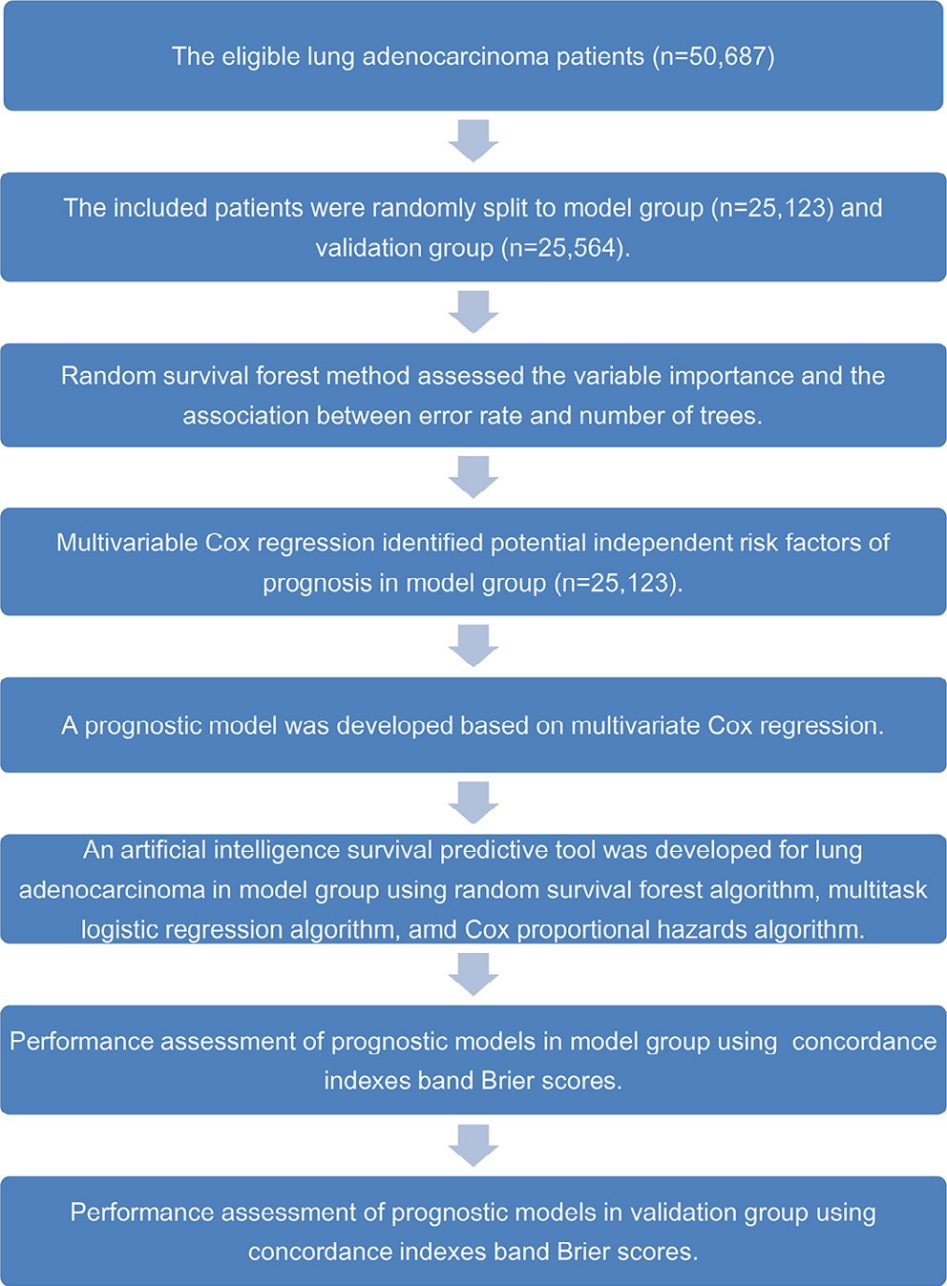
Flow chart of study methodology.

R software 3.5.2 was used to run statistical analysis and relevant algorithms [22], [23], [24], [25], [26], [27], [28], [29]. The research methods and statistical analysis steps were as follows: Continuous data with non normal distribution were presented as median (first and third quantiles). The continuity data were compared using nonparametric test. The counting variables were compared using chi square test. The random survival forest method was used to evaluate variable importance. Multivariate Cox regression was carried out for determining risk factors of LUAD. The prognostic score was constructed according to the coefficient of above risk factors. Kaplan-Meier curve was carried out for presenting prognosis of different cohorts. The area under the time-dependent receiver operating characteristic curve and Brier score were used to assess accuracy of different prognostic models. The flow chart of methodology was presented in Fig. 1.

## 3 Results

### 3.1 Study datasets

The included patients (n = 50,687) were randomly split to model group and validation group. Baseline features for patients in model group and validation group were presented in Table 1. After random grouping, there existed significant differences in mortality, PN, and laterality between model group and validation group, whereas there was no significant difference for other variables between model cohort and validation cohort.

**Table 1:** Clinical features of included patients.

Parameters	Model group	Validation group	Test value	*P* value
Number [n]	25123	25564		
Death [n(%)]	16054(63.9)	16568(64.8)	4.518	0.034
Total survival time (month)	16(5,32)	15(5,32)	323167295	0.107
Age (year)	67(59,75)	67(59,75)	319958070	0.760
Male [(n)%]	13220(52.6)	13478(52.7)	0.048	0.826
Stage 1 [n(%)]	5838(23.2)	5826(22.8)	2.920	0.404
Stage 2 [n(%)]	1902(7.6)	2019(7.9)		
Stage 3 [n(%)]	4494(17.9)	4565(17.9)		
Stage 4 [n(%)]	12889(51.3)	13154(51.5)		
AJCC PT (T0) [n(%)]^#^	140(0.6)	180(0.7)	4.931	0.294
AJCC PT (T1) [n(%)]^#^	6468(25.7)	6563(25.7)		
AJCC PT (T2) [n(%)]^#^	7730(30.8)	7795(30.5)		
AJCC PT (T3) [n(%)]^#^	5097(20.3)	5183(20.3)		
AJCC PT (T4) [n(%)]^#^	5688(22.6)	5843(22.9)		
AJCC PN (N0) [n(%)]^#^	10592(42.2)	10556(41.3)	12.226	0.007
AJCC PN (N1) [n(%)]^#^	2193(8.7)	2342(9.2)		
AJCC PN (N2) [n(%)]^#^	8576(34.1)	8995(35.2)		
AJCC PN (N3) [n(%)]^#^	3762(15.0)	3671(14.4)		
AJCC PM (M0) [n(%)]^#^	12234(48.7)	12410(48.5)	0.111	0.740
AJCC PM (M1) [n(%)]^#^	12889(51.3)	13154(51.5)		
Chemotherapy[n(%)]	11834(47.1)	12056(47.2)	0.014	0.907
Radiation_Surgery[n(%)]	21869(87.0)	22287(87.2)	0.189	0.663
Grade 1[n(%)]	2035(13.2)	2092(13.3)	0.941	0.815
Grade 2[n(%)]	5919(38.5)	6082(38.7)		
Grade 3[n(%)]	7257(47.2)	7396(47.1)		
Grade 4[n(%)]	151(1.0)	139(0.9)		
Laterality(Right)[n(%)]	14647(58.3)	15,084(59.0)	6.401	0.041
Laterality(Left)[n(%)]	9926(39.5)	9865(38.6)		
Laterality(Other)[n(%)]	550(2.2)	615(2.4)		
White [n(%)]	19538(77.9)	19773(77.5)	3.542	0.617
Black[n(%)]	3126(12.5)	3227(12.7)		
American Indian, Aleutian, Alaskan Native [n(%)]	108(0.4)	133(0.5)		
Chinese[n(%)]	591(2.4)	583(2.3)		
Japanese[n(%)]	186(0.7)	193(0.8)		
Other[n(%)]	1534(6.1)	1598(6.3)		
Regional_Nodes_Positive(n)	98(1,98)	98(1,98)	319907291	0.4
Regional_Nodes_Examined(n)	0(0,7)	0(0,7)	321556157.5	0.765
Tumor_Size(mm)	34(21,52)	34(22,52)	269170130.5	0.332

Note: Continuous variables were presented as median (the first quantile, the third quantile); ^#^AJCC: American Joint Committee on Cancer.

### 3.2 Variable importance assessment and selection

Random survival forest method was carried out to assess the variable importance and the association between error rate and number of trees. As shown in Supplementary document 1, the variable importance from high to low was as follows: stage, PM, chemotherapy, PN, age, PT, gender, and radiation_surgery.

Through multivariable Cox regression, stage, PM, chemotherapy, PN, age, PT, sex, and radiation_surgery were identified as independent risk factors of prognosis in model group (Table 2). In validation group, stage, PM, chemotherapy, PN, age, PT, sex, and radiation_surgery were determined as risk factors of LUAD.

**Table 2:** Results of Cox regression analyses.

Variable	Univariate analysis			Multivariate analysis			
	HR^#^	95% CI*	*P*-value	Coefficient	HR^#^	95% CI*	*P*-value
Model cohort (n = 25,123)							
Gender(Male/Female)	1.365	1.323–1.407	<0.001	0.28	1.323	1.283–1.365	<0.001
Age(High/Low)	1.108	1.074–1.142	<0.001	0.244	1.276	1.236–1.317	<0.001
Stage(3–4/1–2)	6.057	5.779–6.349	<0.001	1.286	3.618	3.378–3.875	<0.001
PT(3–4/0–2)	2.209	2.141–2.279	<0.001	0.149	1.16	1.122–1.199	<0.001
PN(1–3/0)	2.825	2.729–2.925	<0.001	0.431	1.539	1.477–1.603	<0.001
PM(1/0)	4.67	4.511–4.835	<0.001	0.937	2.551	2.445–2.662	<0.001
Chemotherapy (Yes/No)	1.362	1.319–1.406	<0.001	−0.726	0.484	0.467–0.501	<0.001
Radiation_Surgery (Yes/No)	0.987	0.944–1.032	0.565	−0.146	0.864	0.824–0.906	<0.001

Validation cohort (n = 25,564)							
Gender(Male/Female)	1.349	1.308–1.391	<0.001	0.247	1.28	1.242–1.320	<0.001
Age(High/Low)	1.113	1.080–1.147	<0.001	0.233	1.262	1.223–1.302	<0.001
Stage(3–4/1–2)	5.688	5.435–5.953	<0.001	1.176	3.24	3.030–3.465	<0.001
PT(3–4/0–2)	2.224	2.156–2.294	<0.001	0.204	1.226	1.186–1.267	<0.001
PN(1–3/0)	2.804	2.709–2.901	<0.001	0.471	1.602	1.537–1.669	<0.001
PM(1/0)	4.502	4.351–4.657	<0.001	0.927	2.528	2.424–2.636	<0.001
Chemotherapy (Yes/No)	1.311	1.271–1.353	<0.001	−0.752	0.471	0.455–0.488	<0.001
Radiation_Surgery (Yes/No)	0.984	0.940–1.029	0.466	−0.123	0.884	0.845–0.926	<0.001

Note: ^#^HR, hazard ratio; *CI, confidence interval.

### 3.3 Prognostic models

Through multivariate Cox regression, a prognostic model was developed with following formula: Prognostic score = (0.786*Stage) + (−0.692*Chemotherapy) + (0.156*PN) + (0.0140*Age) + (0.2684*Gender) + (0.070*PT) + (−0.135*Radiation_Surgery) + (0.091*PM). A RFS model and MTLR model were built to predict the prognosis using eight above prognostic variables identified in the Cox regression.

### 3.4 Artificial intelligence predictive system

**Fig. 2:**
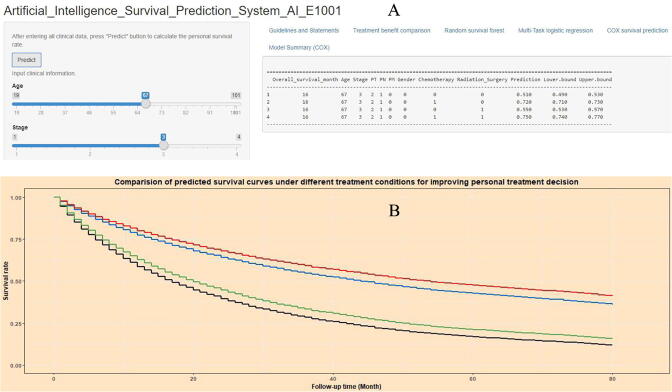
Home page of Artificial intelligence survival prediction system: (A). Data input page and digital result display page; (B).Comparison of predicted survival curves under different treatment conditions.

The current study developed an artificial intelligence survival predictive tool for LUAD (Fig. 2). This tool is available at https://zhangzhiqiao11.shinyapps.io/Artificial_Intelligence_Survival_Prediction_System_AI_E1001/.

The generated survival rate and 95% confidence interval were shown in Fig. 2A. Moreover, Fig. 2B provided comparisons of four predicted survival curves (red, blue, green, and black line in Fig. 2B) under different treatments.

**Fig. 3:**
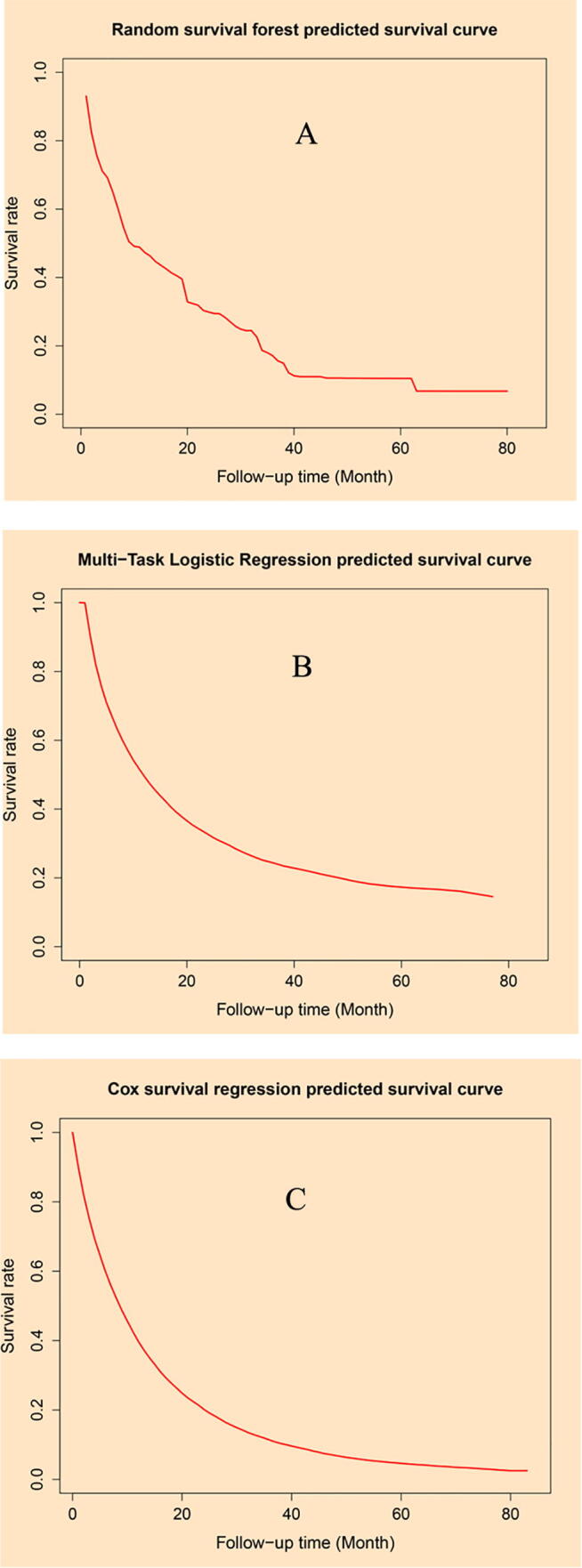
Home page of Artificial intelligence survival prediction system: (A). Predictive personal survival curve by random survival forest, (B). predictive personal survival curve by Multi-task logistic regression; (C). predictive personal survival curve by Cox survival regression.

Three individualized mortality risk curves were presented for RFS (Fig. 3A), MTLR (Fig. 3B), and Cox models (Fig. 3C). The abscissa of each point in the survival curve (Fig. 3) corresponded to a specific time point, and the ordinate represented the predicted survival probability of a special individual patient at that specific time point.

### 3.5 Performance of prognostic models

**Fig. 4:**
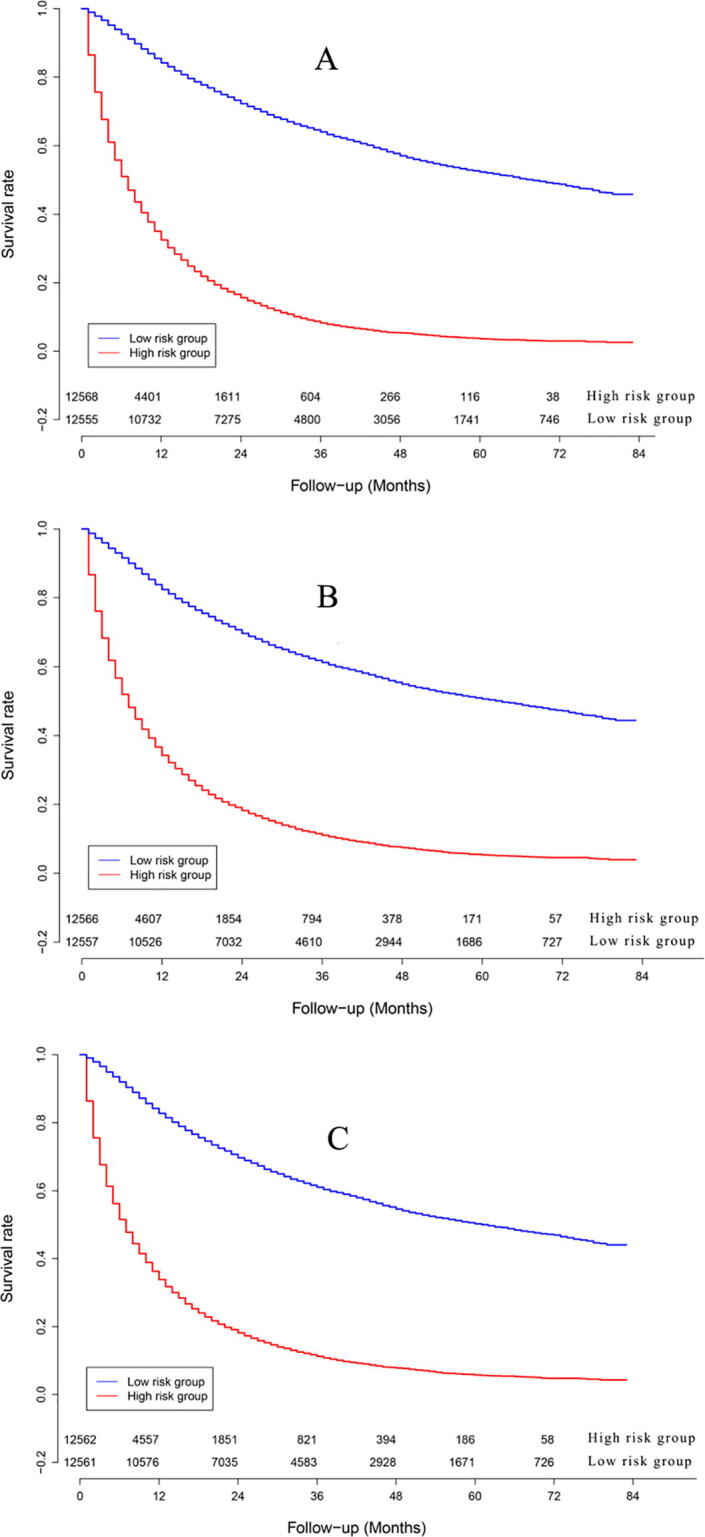
Survival curves of high risk patients and low risk patients in model cohort: (A). Random survival forest: (B). Multi-task logistic regression: (C). Cox survival regression.

The survival curve chart (Fig. 4) indicated that three artificial intelligence prognostic models could discriminate high mortality risk patients from low mortality risk patients in the model cohort.

**Fig. 5:**
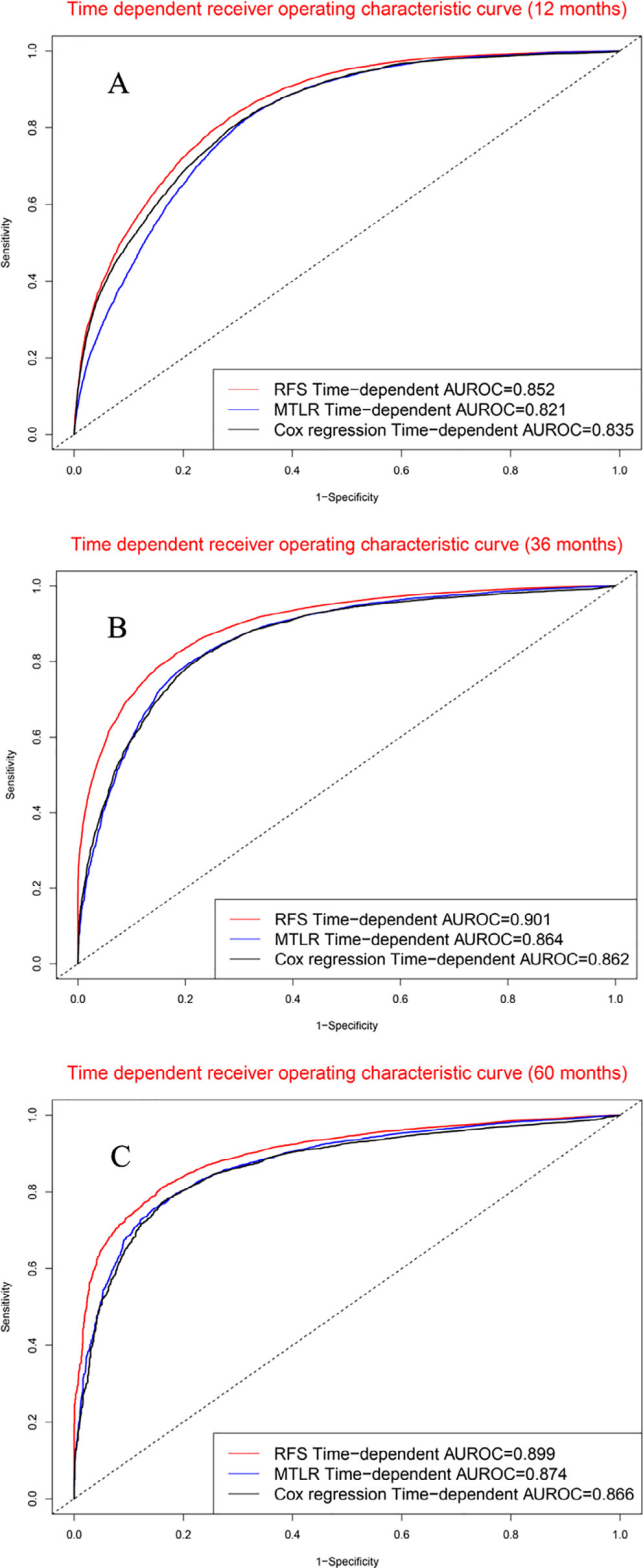
Time-dependent receiver operating characteristic curves in model cohort: (A). 12 months. (B). 36 months, (C). 60 months.

For 12-month survival rate (Fig. 5A), the concordance indexes of RFS, MTLR, and Cox models were 0.852, 0.821, and 0.835, respectively. For 36-month survival rate (Fig. 5B), the concordance indexes of RFS, MTLR, and Cox models were 0.901, 0.864, and 0.862, respectively. For 60-month survival rate (Fig. 5C), the concordance indexes of RFS, MTLR, and Cox models were 0.899, 0.874, and 0.866, respectively.

**Fig. 6:**
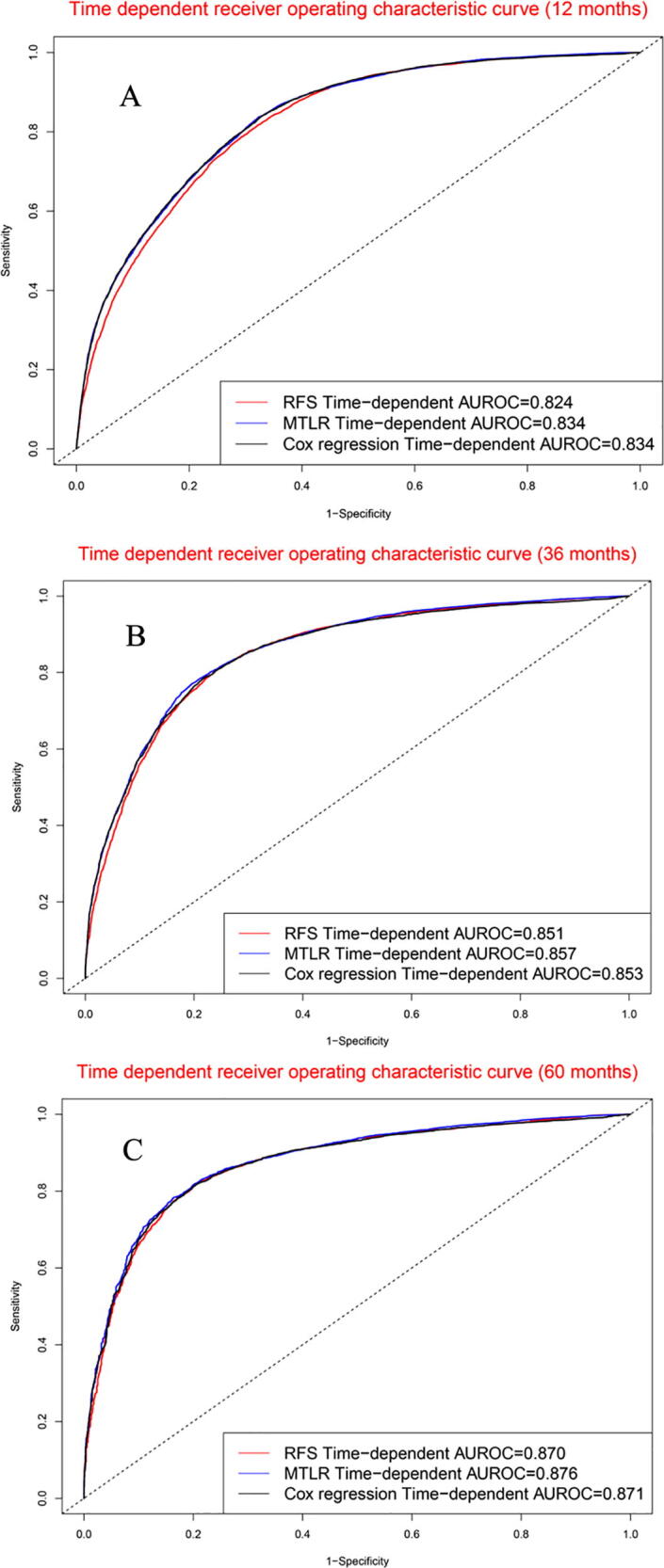
Time-dependent receiver operating characteristic curves in validation cohort: (A). 12 months; (B). 36 months; (C). 60 months.

The survival curve chart (Supplementary document 2) indicated that three artificial intelligence prognostic models could discriminate high mortality risk patients from low mortality risk patients in the validation cohort. For 12-month survival rate (Fig. 6A), the concordance indexes of RFS, MTLR, and Cox model were 0.824, 0.834, and 0.834, respectively. For 36-month survival rate (Fig. 6B), the concordance indexes of RFS, MTLR, and Cox models were 0.851, 0.857, and 0.853, respectively. For 60-month survival rate (Fig. 6C), the concordance indexes of RFS, MTLR, and Cox models were 0.870, 0.876, and 0.871, respectively.

Brier scores of RFS, MTLR, and Cox models were 0.124, 0.152, and 0.143, respectively, indicating that accuracy of RFS Model was better than that of MTLR model and Cox model.

Supplementary document 3, 4, and 5 showed calibration plots of RFS, MTLR, and Cox models in the model cohort. Supplementary document 6, 7, and 8 showed calibration plots of RFS, MTLR, and Cox models in the validation cohort.

## 4 Discussion

The current study established an interesting artificial intelligence survival predictive system for LUAD patients. Three different artificial intelligence algorithms could provide individual survival curves that supported and corroborated one another. More importantly, this artificial intelligence survival predictive system could successfully predict and compare individual mortality risk curves under four treatments, providing clinical benefit comparisons at the individual level to optimize individualized treatment decisions.

The current study provides a convenient individual mortality risk predictive tool for lung adenocarcinoma patients. For example, the 16-month(user selected time-point) survival rate were 0.51 for non-treatment status (black line in Fig. 2), 0.72 for chemotherapy status (blue line in Fig. 2), 0.55 for radiation_surgery status (green line in Fig. 2), and 0.75 for combination therapy status (red line in Fig. 2) for a special patients with the following parameters: age 67 years, stage 3, PT 2, PN 1,PM 0, and gender female. Through the predicted survival rate at a specific time-point in the upper part of Fig. 2 and the individual survival curve in the lower part of Fig. 2, patients can easily get their own individual survival curve, so as to optimize individualized treatment decisions.

Several predictive models were constructed for predicting overall survival for lung cancer at the group level [3], [4], [22]. However, these prognostic models could only forecast mortality risk at group level with unique clinical features. Our predictive system could provide individualized survival curves at individual level, which is importance for individualized treatment decisions. Additionally, our survival predictive system predicted and compared the individual survival curves under different treatments, which is valuable for patients to make optimal medical decisions before treatment.

Considering the opaque nature of the operation process of artificial intelligence algorithms, the current research provided three individual survival curves predicted using different artificial intelligence algorithms for clinical application. The concordances of prognostic models based on MTLR, RFS, and Cox algorithms were 0.703, 0.650, and 0.698, respectively, for glioblastoma multiforme patients (the higher the concordance, the higher the accuracy of the prognostic model), whereas the Brier scores were 0.039, 0.059, and 0.040, respectively, (the smaller the Brier score, the higher the accuracy of the prognostic model) [39], indicating that MTLR algorithm was superior to RFS and Cox algorithms for prognostic prediction. The MTLR model had an AUROC of 0.92 and a brier score of 0.08, suggesting good clinical application value for prognostic prediction [36]. The RSF algorithm performed better than Cox algorithm for predicting the prognosis of major adverse cardiac and cerebrovascular event patients [40]. Harvard University artificial intelligence research team developed an artificial intelligence predictive tool for predicting prognosis of glioblastoma and provided individual predictive information of predicted survival time, one-year survival rate, and overall survival curves [41]. The concordances of prognostic models based on RFS and Cox algorithms were 0.680 and 0.690, respectively, for glioblastoma patients in another prognostic study [41]. Combined with the concordance indexes of different algorithms in the current research and the conclusions of previous studies, we first recommended the survival curve predicted by MTLR algorithm, and the survival curves predicted by RFS algorithm and Cox algorithm might be used as the second and third recommendation survival predicted curves.

Random survival forest algorithm has the following abilities: dealing with multicollinearity effects, selecting the most important parameters in accordance with defined tree threshold, and assessing the variable relative importance [42], [43]. The RFS algorithm has been recommended for prognostic models and was reported to be superior to the Cox model in terms of predictive accuracy [44], [45], [46]. It was reported that multitask learning algorithm was superior to Cox algorithm in cancer survival analysis [47]. The concordance indexes and calibration plots of the RFS and MTLR models indicated good predictive performance, which was similar to that of the Cox model. RFS model scored higher than Cox and MTLR models on Brier score. Our results indicated that the RFS and MTLR models have good clinical application values, which were not inferior to the Cox model in survival analysis.

Our artificial intelligence predictive tool could predict the survival curve of lung cancer patients under different treatments and its 95% confidence interval. An individualized survival predictive function is very important for identifying patients at high mortality risk. Our artificial intelligence predictive tool is helpful for providing valuable predictive information for individual patient survival rate in optimizing the comprehensive management and individualized treatment for LUAD patients. In clinical work, it is necessary for lung cancer patients who were predicted as high mortality risk by artificial intelligence predictive tool to consider receiving more active and timely antitumor treatment to improve the prognosis.

It was true that artificial intelligence methods have made great progress in the fields of tumor diagnosis, treatment, prognostic prediction, and research. However, in the clinical field, artificial intelligence method can never become a substitute for professional medical personnel, but exists as assistants and tools of medical personnel. Medical treatment is a comprehensive prevention and treatment system covering physical, psychological, and social relations, rather than a simple superposition of high-end equipment, cold technology, and complex algorithms. For clinicians, artificial intelligence technology helps to optimize the diagnosis and treatment of tumors.

Limitations: First, because lung cancer has high clinical heterogeneity, the treatment of lung cancer was too complex to form a unified treatment. Meanwhile, the great progress of radiotherapy, chemotherapy, and surgery were not conducive to forming the clinical subgroups. Although the SEER database provided limited treatment information (including radiotherapy, chemotherapy, and surgery), the treatment information was not sufficient to divide patients into stable subgroups. Second, all study patients were included from 2010 to 2015, resulting in a relatively short follow-up time (minimum follow-up time: 36 months; maximum follow-up time: 83 months). A long follow-up time is valuable for ascertaining the clinical application value of survival predictive system for long time points of greater than 83 months. Third, as nonparametric algorithms, the RFS and MTLR algorithms couldn’t be directly expressed by conventional mathematical formulas, weakening the interpretability and clinical application of these predictive models to a certain extent. Fourth, external research datasets provide more convincing evidences for the conclusions of prognostic studies. However, we failed to identify a long-term tumour research dataset similar to the dataset provided by the SEER database. Independent external follow-up datasets are of great value for the construction and verification of tumour prognostic research.

## 5 Conclusions

The current study designed an individualized survival predictive system, which could provide individual survival curves using three different artificial intelligence algorithms. This artificial intelligence predictive system could directly convey treatment benefits by comparing individual mortality risk curves under different treatments. This artificial intelligence predictive tool is available at https://zhangzhiqiao11.shinyapps.io/Artificial_Intelligence_Survival_Prediction_System_AI_E1001/.

## Ethics approval

The current study was approved by ethics committee of Shunde Hospital, Southern Medical University and exempted from informed consent.

## Consent for publication

All authors have reviewed the manuscript and consented for publication.

## Availability of data and materials

The study data are available at the SEER database (https://seer.cancer.gov/).

## Funding

This study was supported by 10.13039/501100011478Foshan Science and Technology Bureau (2020001004584). The funders had no role in study design, data collection and analysis, decision to publish, or preparation of the manuscript.

## CRediT authorship contribution statement

**Tingshan He:** Conceptualization, Methodology, Resources, Investigation, Data curation, Formal analysis, Validation, Software, Project administration, Supervision. **Jing Li:** Conceptualization, Methodology, Resources, Investigation, Data curation, Formal analysis, Validation, Software, Project administration, Supervision. **Peng Wang:** Conceptualization, Methodology, Resources, Investigation, Data curation, Formal analysis, Validation, Software, Project administration, Supervision, Visualization. **Zhiqiao Zhang:** Conceptualization, Methodology, Resources, Investigation, Data curation, Formal analysis, Validation, Software, Project administration, Supervision, Visualization, Funding acquisition.

## Declaration of Competing Interest

The authors declare that they have no known competing financial interests or personal relationships that could have appeared to influence the work reported in this paper.
