# Supplementary material for: Artificial intelligence predictive system of individual survival rate for lung adenocarcinoma
Source: Comput Struct Biotechnol J. 2022 May 14;20:2352–9. doi: 10.1016/j.csbj.2022.05.005 (PMC9123088; doi:10.1016/j.csbj.2022.05.005)
Supplement: Supplementary data 1 [file mmc1.pdf]

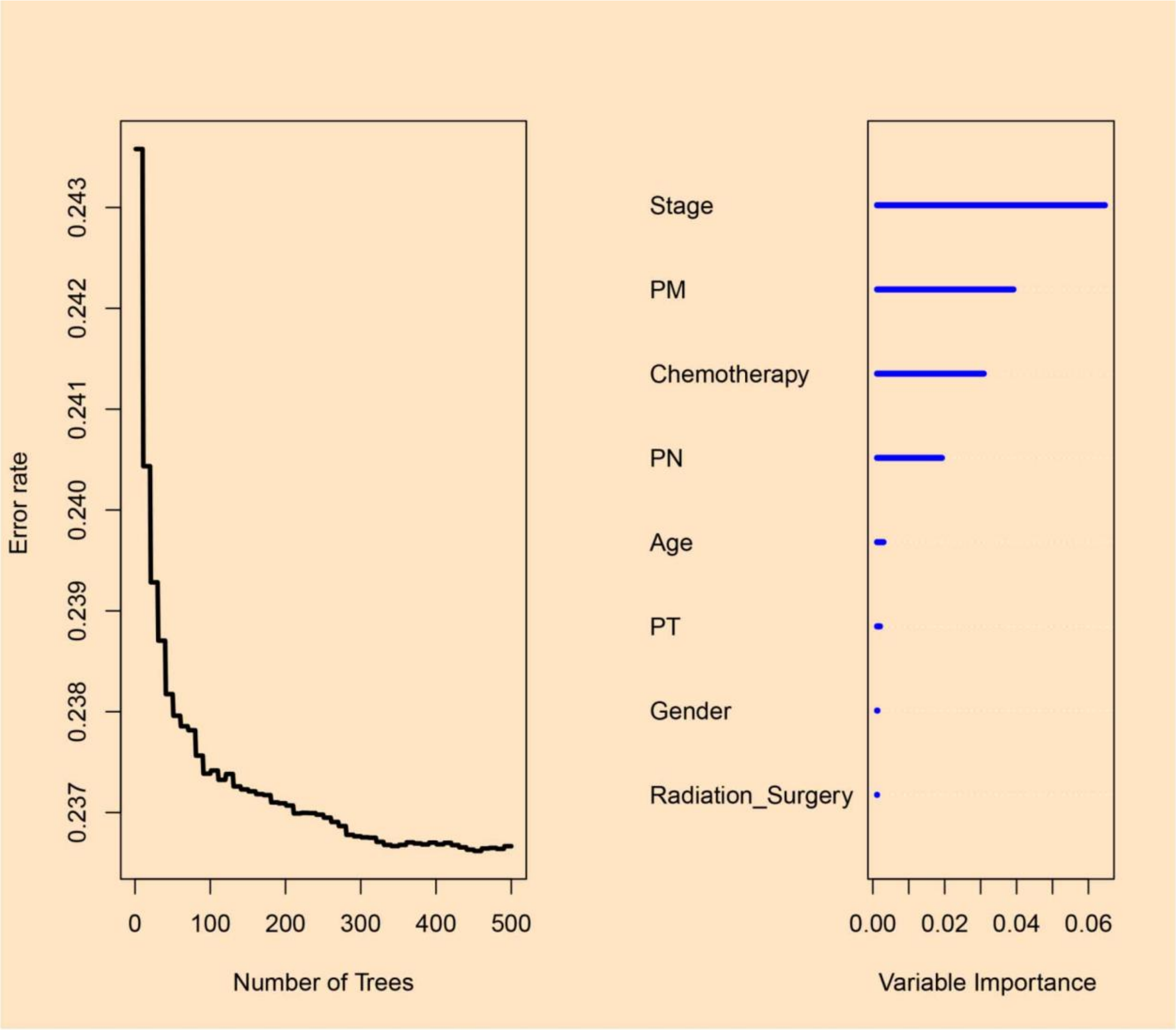

Supplementary Figure 1. Error rate chart and variable importance assessment chart in random survival forest algorithm.

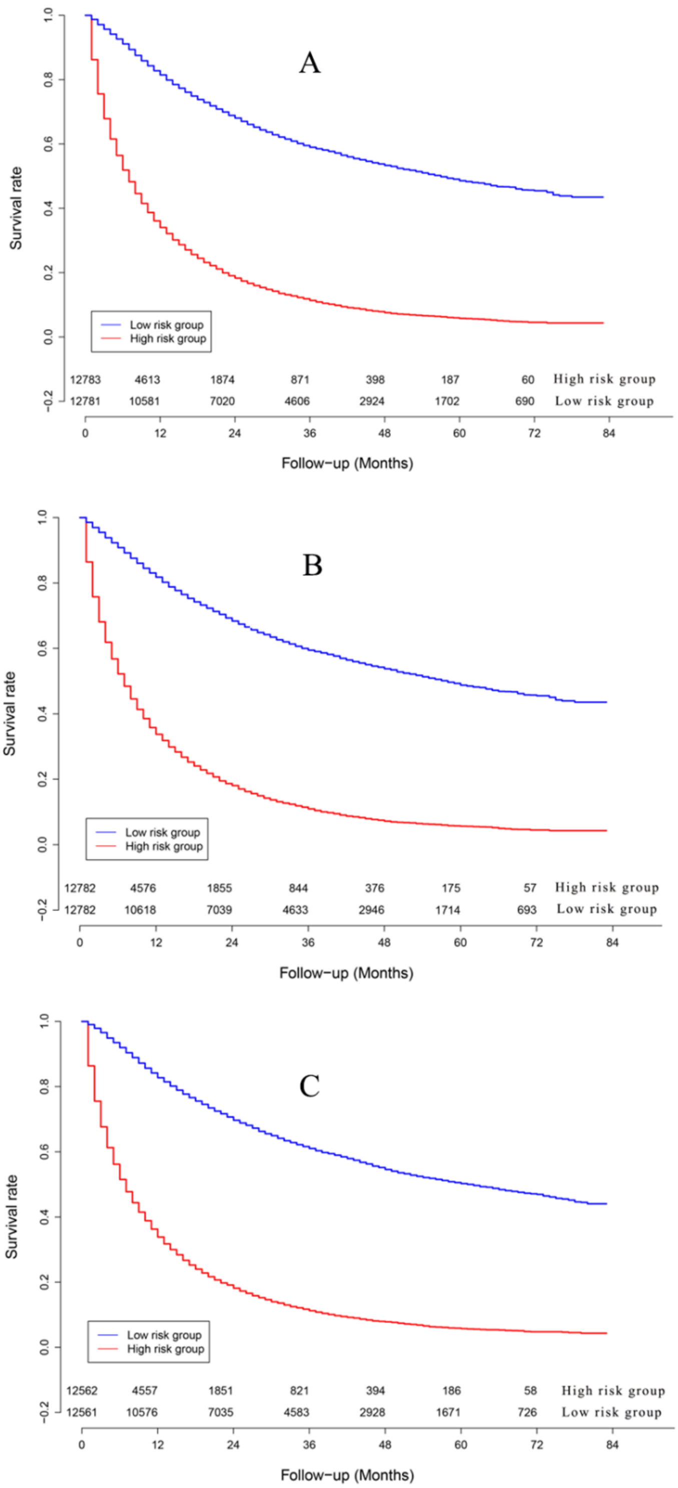

Supplementary Figure 2. Survival curves of high risk patients and low risk patients in validation cohort: (A). Random survival forest; (B). Multi-task logistic regression; (C). Cox survival regression.

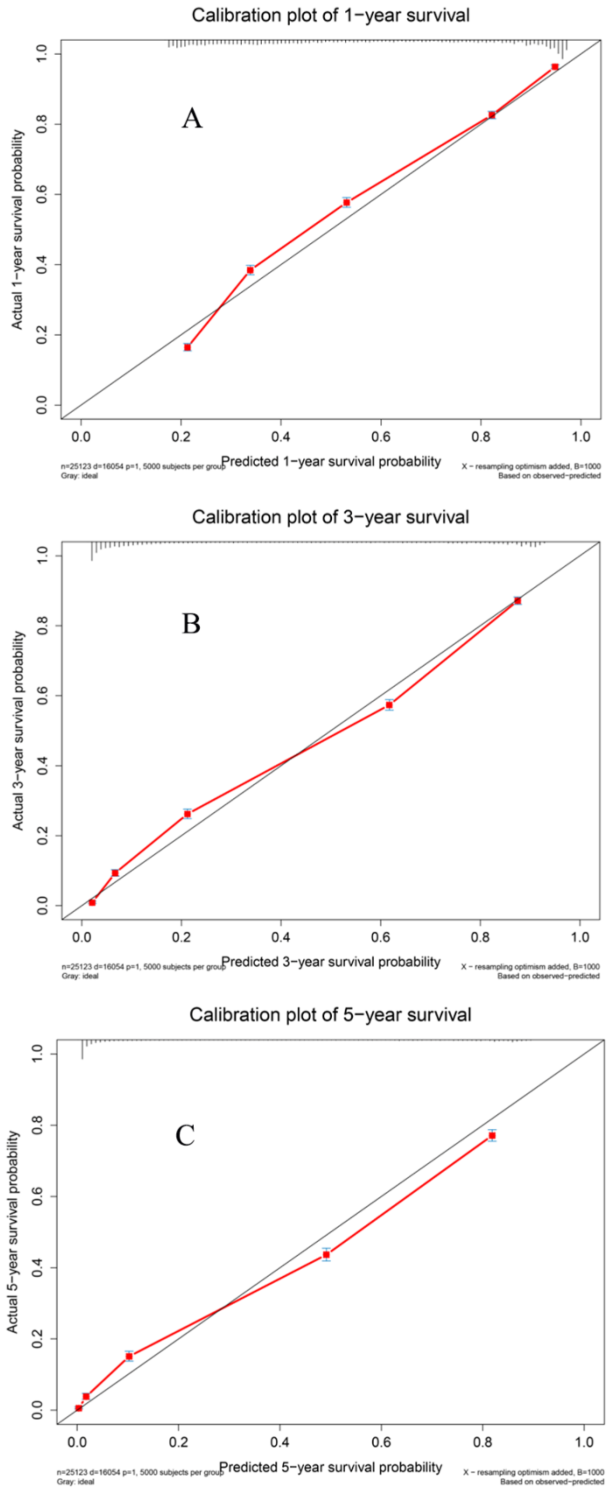

Supplementary Figure 3. Calibration curves of Random survival forest in model cohort: (A). Calibration curve for 1-year survival; (B). Calibration curve for 3-year survival; (C). Calibration curve for 5-year survival.

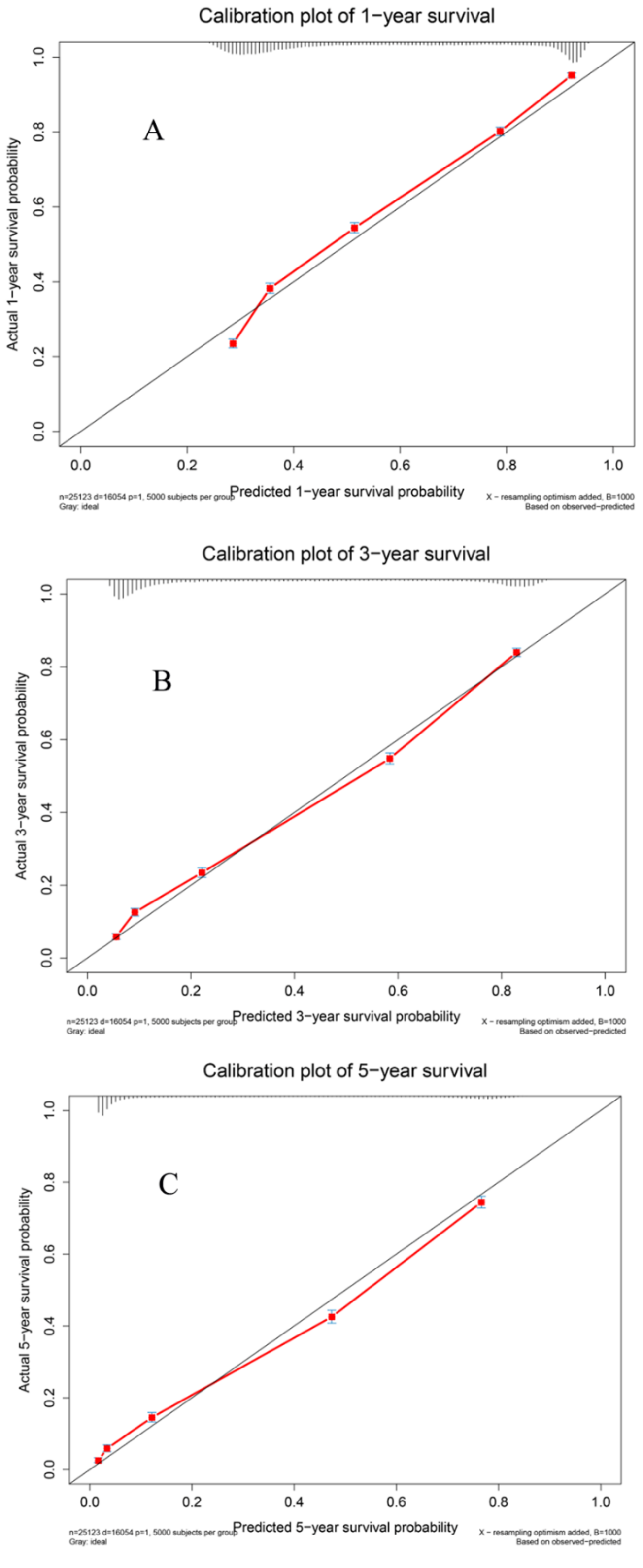

Supplementary Figure 4. Calibration curves of Multi-task logistic regression in model cohort: (A). Calibration curve for 1-year survival; (B). Calibration curve for 3-year survival; (C). Calibration curve for 5-year survival.

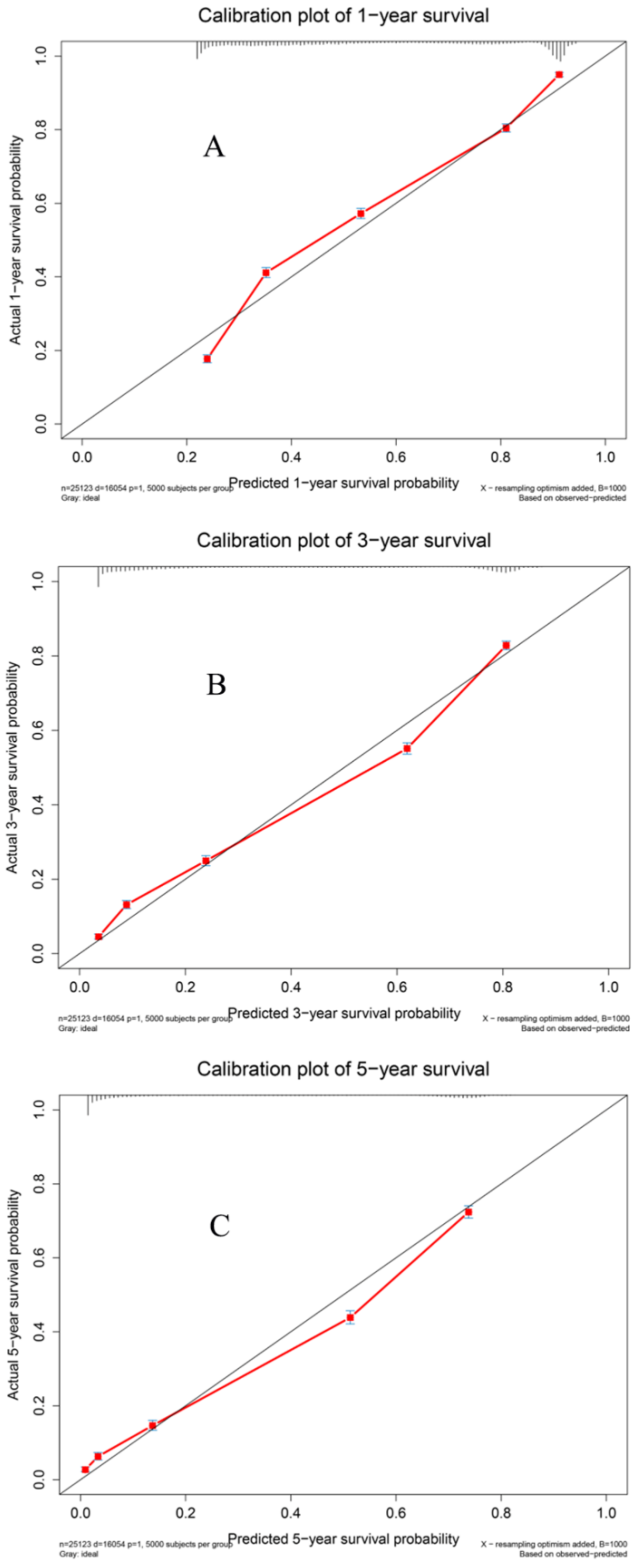

Supplementary Figure 5. Calibration curves of Cox survival regression in model cohort: (A). Calibration curve for 1-year survival; (B). Calibration curve for 3-year survival; (C). Calibration curve for 5-year survival.

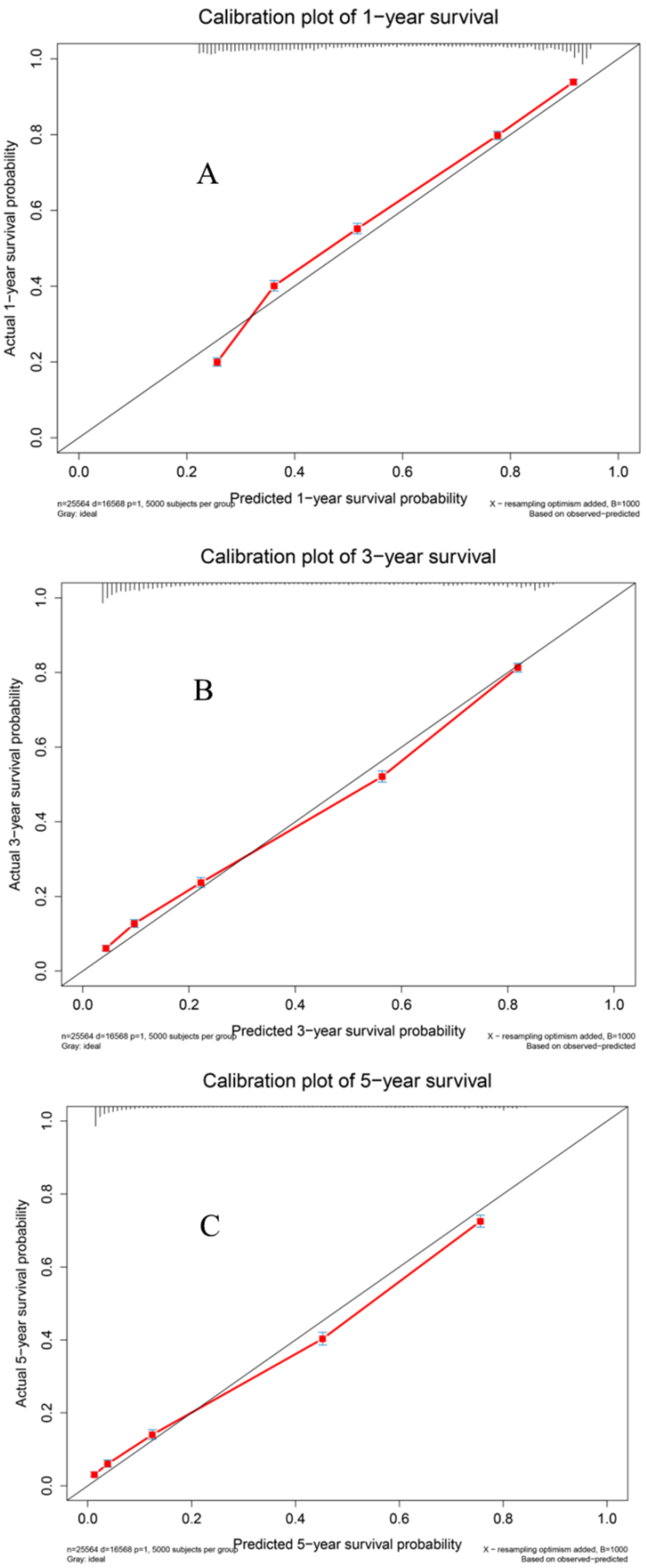

Supplementary Figure 6. Calibration curves of Random survival forest in validation cohort: (A). Calibration curve for 1-year survival; (B). Calibration curve for 3-year survival; (C). Calibration curve for 5-year survival.

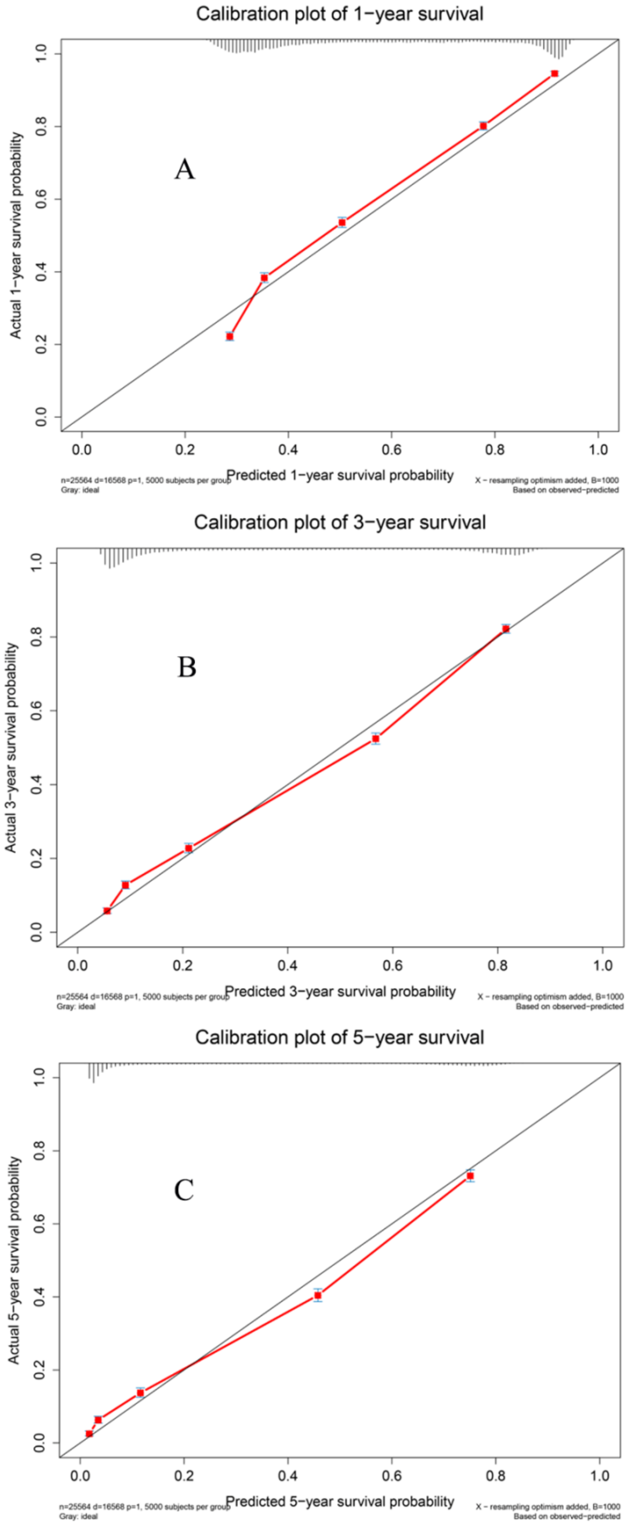

Supplementary Figure 7. Calibration curves of Multi-task logistic regression in validation cohort: (A). Calibration curve for 1-year survival; (B). Calibration curve for 3-year survival; (C). Calibration curve for 5-year survival.

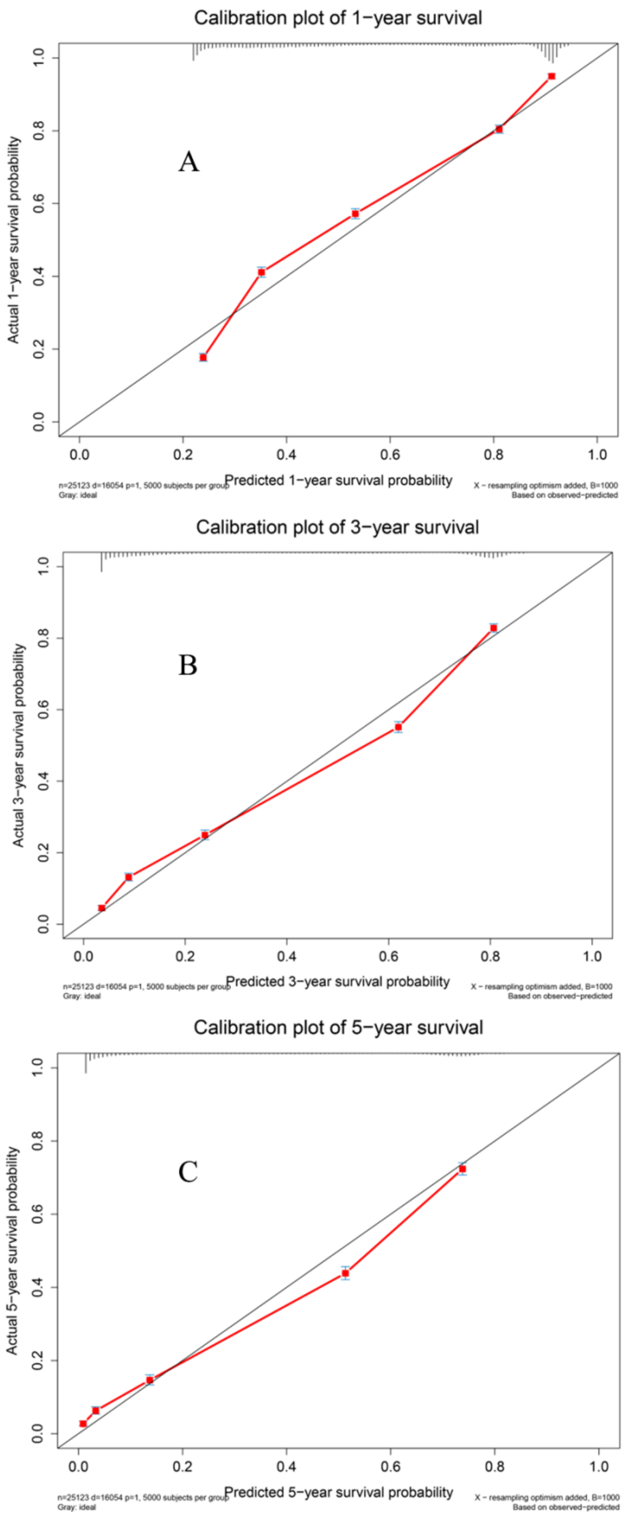

Supplementary Figure 8. Calibration curves of Cox survival regression in validation cohort: (A). Calibration curve for 1-year survival; (B). Calibration curve for 3-year survival; (C). Calibration curve for 5-year survival.
